# A multimedia dataset for object-centric business process mining in IT asset management

**DOI:** 10.1016/j.dib.2024.110716

**Published:** 2024-07-08

**Authors:** Diana Chvirova, Andreas Egger, Tobias Fehrer, Wolfgang Kratsch, Maximilian Röglinger, Jakob Wittmann, Niklas Wördehoff

**Affiliations:** a Branch Business & Information Systems Engineering of the Fraunhofer FIT, Alter Postweg 101, D-86159 Augsburg, Germany; b Branch Business & Information Systems Engineering of the Fraunhofer FIT, Wittelsbacherring 10, D-95444 Bayreuth, Germany; c FIM Research Center for Information Management, Alter Postweg 101, D-86159 Augsburg, Germany

**Keywords:** Process mining, Video mining, Business process management, Object-centric event log

## Abstract

This manuscript introduces a multimedia business process dataset provided by a German research institute. The dataset was systematically collected in a laboratory environment that reflects the workspace of IT staff managing IT Asset Management (ITAM) processes. It encompasses data from 121 process instances across six basic processes, captured using 37 video recordings from two camera perspectives, motion tracking, environmental sensors, an ITAM system trace, and event log data from user interactions. The data is made available in its raw state and processed form. The object-centric event log format (OCEL) provides discrete business process events from system activities. Event data from reality is supplied as raw video files and logs from environmental sensors. The video files were also manually labelled with identifiable business process activities and their associated entities. This multimedia dataset has been designed as a resource for developing, training, and evaluating process mining techniques based on unstructured data. Consequently, the dataset design emphasizes the traceability of activities and entities across the multimedia data sources.

Specifications Table

**Table:** 

Subject	Data Science: Data Mining and Statistical Analysis Computer Science: Computer Vision and Pattern Recognition
Specific subject area	Ground truth data, event log data, and sensor data are required to build, train, and evaluate approaches for the automatic computation of enriched process mining event logs.
Type of data	Video, Image, Structured Text (JSON, XML, CSV), Graph, Figure, Raw, Analyzed, Filtered, Processed.
Data collection	Data were collected in a lab environment that resembles the work environment of IT staff overseeing IT asset management. A total of 36 scripted scenes were recorded from two camera angles. Additional sensors measure actor movement, gestures, and location in the room. Further ambient sensors monitor the environment (e.g., door openings). An IT asset management system stores business process activities as performed by IT staff and their clients.
Data source location	Video recordings were collected at the institution location in Augsburg, Germany (48° 20’ 16.6956” N, 10° 54’ 1.836” E). Data are stored at University of Bayreuth, Bayreuth, Germany.
Data accessibility	Repository name: Business Processes in IT Asset Management Multimedia Event Log Data identification number: 10.6084/m9.figshare.25246291 Direct URL to data: https://figshare.com/articles/dataset/Business_Processes_in_IT_Asset_Management_Multimedia_Event_Log/25246291

## 1 Value of the Data

• Based on a systematic data collection setup, this data set provides coherent data from executing 121 process instances in IT asset management.
• By integrating data from various sources, including IT system logs, video cameras, and ambient sensors, the dataset provides a multimedia view of process execution. This fusion of media streams allows researchers to explore correlations between system-generated events and real-world activities.
• The alignment of system-generated event data with observational data from workplace environments bridges the gap between IT system behaviour and physical actions. Researchers can investigate how system events manifest in practical scenarios.
• The dataset offers both raw and processed data. System event logs follow the OCEL 1.0 format, while real-life event data includes video recordings and sensor logs. Video data was manually labelled with segments of business process activities.
• Fellow researchers are encouraged to leverage this dataset for developing, training, and evaluating process mining techniques. The data in structured and unstructured formats allow researchers to move away from the labelled ground truth and to analyze the data according to their specific focus.
• Questions and problems for future research that might benefit from the dataset include the development of abstraction techniques to identify process activities from low-level events, the consideration of uncertainty in events or data, and searching for techniques that aim to enrich process data from unstructured data sources gradually.

## 2 Background

Process mining encompasses a range of individual techniques that facilitate various analyses, including process discovery, monitoring, and process improvement based on historical process execution data [1]. These techniques rely on traces of process event data, primarily extracted from information systems such as ERP systems. While these systems capture many aspects of critical business processes, there remain significant blind spots in process activities. A growing research stream aims to address these blind spots by developing approaches illuminating previously unexplored areas—specifically, leveraging distributed, unstructured data sources. These alternative event data sources include audio, image/video, text, sensor data, and raw system logs [[2], [3], [4], [5]]. To advance these approaches, researchers seek well-documented, annotated raw data that provides insights into process behaviour, the entities involved, and the relationships across different data sources. An ideal domain for studying business processes involving both in-system and out-of-system data is IT asset management (ITAM). In ITAM, staff oversee the entire lifecycle of assets within IT systems while managing physical objects in a controlled and straightforward environment.

## 3 Data Description

**Fig. 1:**
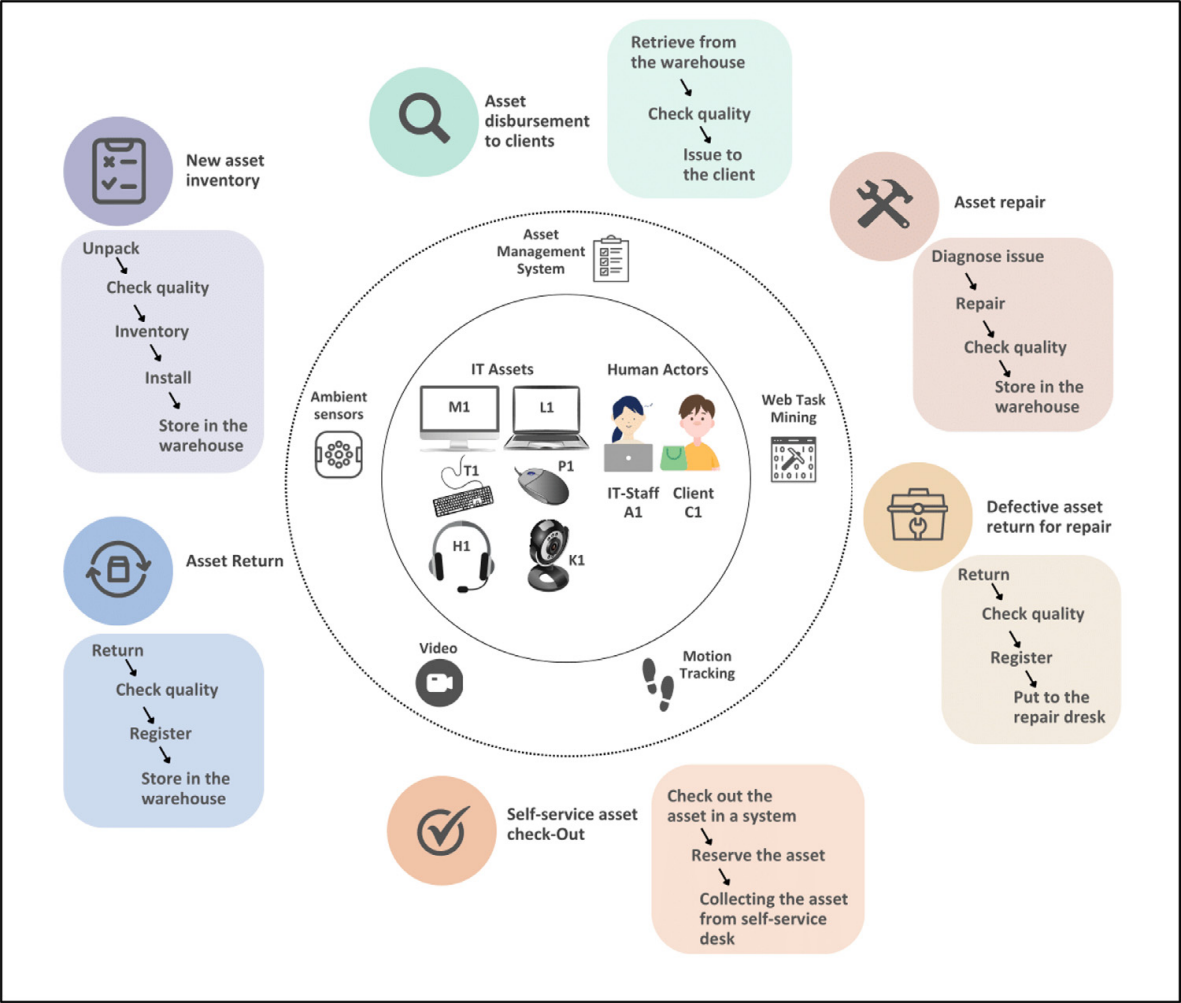
Overview of data collection.

The *Business Processes in IT Asset Management Multimedia Event* Log dataset [6] comprises 121 prescripted business process instances of six baseline processes in ITAM. The process instances were recorded in 36 scenes in a controlled laboratory environment for data collection. Each scene contains multiple completed process instances that may partially or fully overlap within one scene. The process environment simulates that of a small IT department overseeing ITAM processes. Within this context, IT staff diligently manage IT assets while clients interact with them—whether by collecting or returning them for repair. The dataset includes various IT assets like laptops, monitors, and keyboards. Fig. 1 gives an overview, and the following descriptions outline the process types, recorded scenes, and corresponding process instances, as well as detail the dataset files. For further exploration, an interactive dataset documentation is accessible online^1^ and linked to the data repository.

## 4 ITAM Process Overview

1. *New asset inventory.* IT staff integrates new assets into the existing inventory framework. Assets must be unpacked, tested, inventoried in the ITAM system, installed, and stored in the warehouse.
2. *Asset disbursement to clients*. IT staff manage inventory issuance using the ITAM system, recording transactions and client acceptances. In the warehouse, assets are retrieved, inspected for quality, and issued to clients.
3. *Asset repair*. IT staff identify and diagnose asset issues at the repair desk. The asset is tested after thorough quality checks and necessary repairs to ensure full functionality. The repaired asset is then updated in the ITAM system and returned to the warehouse.
4. *Defective asset return for repair*. IT staff processes the return of defective assets that need repair. The process starts at the IT working desk, where IT staff document the transaction, perform a thorough quality assessment, and check the asset into the ITAM system to ensure it is logged as awaiting repair. The asset is then moved to the repair desk for further handling.
5. *Asset return.* The asset return process involves logging returned assets into the ITAM system and conducting physical inspections to ensure quality. After verification checks, assets are stored appropriately in the warehouse.
6. *Self-service asset check-out*. This automated process allows clients to independently check out rental assets from the IT inventory via the ITAM system, enhancing convenience and efficiency. Clients access the ITAM system to verify asset availability and reserve their choice, preventing double bookings. After reservation, clients collect their assets from the self-service storage and complete the transaction without needing direct assistance from IT staff.

### 4.1 Environment and objects

The recorded data was collected within a controlled environment, specifically an office room divided into distinct areas (see Fig. 3). To facilitate cross-data-source correlation, all human actors and objects featured in the scenes were assigned types and often artificial IDs. Table 2 provides details of the environments and objects utilized throughout the dataset.

**Table 1:** General dataset properties.

Property	Value
Process Types	6
Event Types	41
Object Types	18
Events	1464
Objects	86
Scenes	36
Process Instances	121

**Table 2:** Listing of environment locations and objects referenced across the dataset.

Location/Object	Abbreviation	Description
**Locations (see**Fig. 3**)**		
IT working desk	–	The desk where the IT staff makes changes in the ITAM system and receives or hands out assets from and to users. The desk is located in the issue area.
Repair desk	–	The desk in the working area where asset repairs are carried out.
Self-service storage	–	The storage in the self-service area near the entrance, where clients can pick up smaller assets themselves.
Warehouse		The location ‘Warehouse’ comprises four storage locations: laptop shelf, mouse and keyboard cupboards, and monitor storage.
Laptop shelf	–	The shelf in the warehouse area where laptops are stored in a stack.
Mouse cupboard	–	The (left) cupboard in the warehouse area where computer mouses are stored.
Keyboard cupboard	–	The (right) cupboard in the warehouse area where keyboards are stored.
Monitor storage		The table in the warehouse area where monitors are stored.
Door		The entrance door - located in the self-service area.
Window		The window where the temperature and humidity sensor is installed. The window is located in the issue area.
**Human Actors**		
IT Staff	A1, A2	Technicians in the IT department manage IT assets and client requests.
Clients	C1-C10	Clients consuming ITAM services.
**IT Assets**		
Laptops	L1-L9, L17-L18, L20	Laptops are each labeled with their ID attached to the device surface.
Monitors	M1-M9	The monitors are located on the table with monitors. All monitors are labeled with their ID on the device front.
Keyboards	T1-T7	The keyboards are located in the keyboard cupboard (the right one on the video). The keyboards are not labeled.
Computer Mouses	P1-P9	The computer mice are located in the mouse cupboard (the left one on the video). The mice are not labeled.
Headsets	H1-H11	The headsets are located on the self-service desk and can be requested via self-service.
Webcams	K1-K8	The webcams are located on the self-service desk and can be requested via self-service.

### 4.2 Scenes and process instances

The dataset is organized at a scene-by-scene level. Table 3 presents the 36 distinct scenes in the overview, each paired with its corresponding set of process instances. Notably, all these scenes were recorded within a single day on 25.03.2024.

**Table 3:** Scenes and processes.

Scene	Process Instances	Scene	Process Instances
Scene01: 08:48:23 (6 m 32 s)	(ID001) Asset disbursement to clients	Scene19 13:36:34 (5 m 23 s)	(ID060) Asset return
	(ID002) Asset disbursement to clients		(ID061) Asset return
	(ID003) Asset disbursement to clients		(ID062) Asset return
Scene02: 08:57:06 (3 m 41 s)	(ID004) New asset inventory		(ID063) Asset disbursement to clients
	(ID005) New asset inventory	Scene20 13:44:10 (8 m 32 s)	(ID065) Asset repair
Scene03: 09:06:07 (12 m 40 s)	(ID006) New asset inventory		(ID066) Defective asset return for repair
	(ID007) Defective asset return for repair		(ID067) Asset repair
	(ID008) Self-service asset check-out		(ID068) Self-service asset check-out
	(ID009) Self-service asset check-out	Scene21 13:55:14 (7 m 4 s)	(ID069) Asset disbursement to clients
Scene04: 09:19:59 (3 m 20 s)	(ID010) New asset inventory		(ID070) Asset disbursement to clients
	(ID011) New asset inventory		(ID071) Asset disbursement to clients
	(ID012) Asset return		(ID072) Self-service asset check-out
Scene05: 09:24:15 (3 m 20 s)	(ID013) Asset repair	Scene22 14:04:10 (7 m 4 s)	(ID073) New asset inventory
	(ID014) Self-service asset check-out		(ID074) Self-service asset check-out
Scene06: 09:29:43 (7 m 12 s)	(ID015) New asset inventory		(ID075) Asset return
	(ID016) Self-service asset check-out	Scene23 14:58:46 (4 m 40 s)	(ID076) Asset disbursement to clients
	(ID017) Asset return		(ID077) Self-service asset check-out
	(ID018) Self-service asset check-out		(ID078) Asset disbursement to clients
Scene07 09:39:03 (6 m 40 s)	(ID019) New asset inventory	Scene24 15:05:42 (10 m 16 s)	(ID079) New asset inventory
	(ID020) New asset inventory		(ID080) Defective asset return for repair
	(ID021) Asset disbursement to clients		(ID081) Asset return
Scene08 09:49:03 (7 m 7 s)	(ID022) Defective asset return for repair	Scene25 15:17:58 (5 m 20 s)	(ID082) New asset inventory
	(ID023) Defective asset return for repair		(ID083) New asset inventory
	(ID024) Asset return	Scene26 15:25:34 (5 m 28 s)	(ID084) Defective asset return for repair
Scene09 10:45:00 (9 m 28 s)	(ID025) Asset disbursement to clients		(ID085) Asset disbursement to clients
	(ID026) Asset disbursement to clients		(ID086) Asset return
	(ID027) Asset disbursement to clients		(ID087) Self-service asset check-out
Scene10 10:58:04 (8 m 0 s)	(ID028) New asset inventory	Scene27 15:33:10 (6 m 15 s)	(ID088) Defective asset return for repair
	(ID029) New asset inventory		(ID089) Self-service asset check-out
	(ID030) Asset repair		(ID090) Asset repair
Scene11 11:08:28 (7 m 52 s)	(ID031) Defective asset return for repair	Scene28 15:42:21 (4 m 48 s)	(ID091) New asset inventory
	(ID032) Asset repair		(ID092) Defective asset return for repair
	(ID033) Self-service asset check-out		(ID093) Asset repair
	(ID034) Self-service asset check-out	Scene29 16:40:27 (3 m 36 s)	(ID094) Asset disbursement to clients
Scene12 11:19:40 (8 m 16 s)	(ID035) Asset disbursement to clients		(ID095) Self-service asset check-out
	(ID036) Asset disbursement to clients		(ID096) Self-service asset check-out
	(ID037) Asset return	Scene30 16:45:07 (3 m 12 s)	(ID097) Asset return
	(ID038) Asset return		(ID098) Asset return
Scene13 11:31:32 (12 m 29 s)	(ID039) Asset disbursement to clients		(ID099) Asset return
	(ID040) Asset disbursement to clients	Scene31 16:49:31 (8 m 53 s)	(ID100) Defective asset return for rep.
	(ID041) Defective asset return for repair		(ID101) Asset return
Scene14 11:46:52 (7 m 8 s)	(ID042) Defective asset return for repair		(ID102) Asset return
	(ID043) Asset repair		(ID103) Asset repair
	(ID044) Self-service asset check-out	Scene32 16:59:15 (5 m 12 s)	(ID104) Asset disbursement to clients
Scene15 11:55:41 (6 m 31 s)	(ID045) Asset disbursement to clients		(ID105) Self-service asset check-out
	(ID046) Asset repair		(ID106) Self-service asset check-out
	(ID047) Asset disbursement to clients	Scene33 17:06:35 (4 m 56 s)	(ID107) Asset return
	(ID048) Asset return		(ID108) Asset return
Scene16 13:14:59 (6 m 16 s)	(ID049) Asset disbursement to clients		(ID109) Asset return
	(ID050) Self-service asset check-out	Scene34 17:14:27 (5 m 44 s)	(ID110) Asset disbursement to clients
	(ID051) Asset disbursement to clients		(ID111) Asset disbursement to clients
	(ID052) Defective asset return for repair		(ID112) Asset disbursement to clients
Scene17 13:22:10 (4 m 56 s)	(ID053) New asset inventory	Scene35 17:27:23 (5 m 20 s)	(ID113) Defective asset return for rep.
	(ID054) New asset inventory		(ID114) Asset disbursement to clients
	(ID055) Defective asset return for repair		(ID115) Self-service asset check-out
Scene18 13:29:06 (5 m 28 s)	(ID056) Asset repair		(ID116) Self-service asset check-out
	(ID057) Asset repair	Scene36 17:35:23 (7 m 12 s)	(ID117) Asset return
	(ID058) Asset disbursement to clients		(ID118) Asset return
	(ID059) Self-service asset check-out		(ID119) Asset return
Scene19 13:36:34 (5 m 23 s)	(ID060) Asset return		(ID120) Asset return
	(ID061) Asset return		(ID121) Asset return
	(ID062) Asset return		
	(ID063) Asset disbursement to clients		

**Fig. 2:**
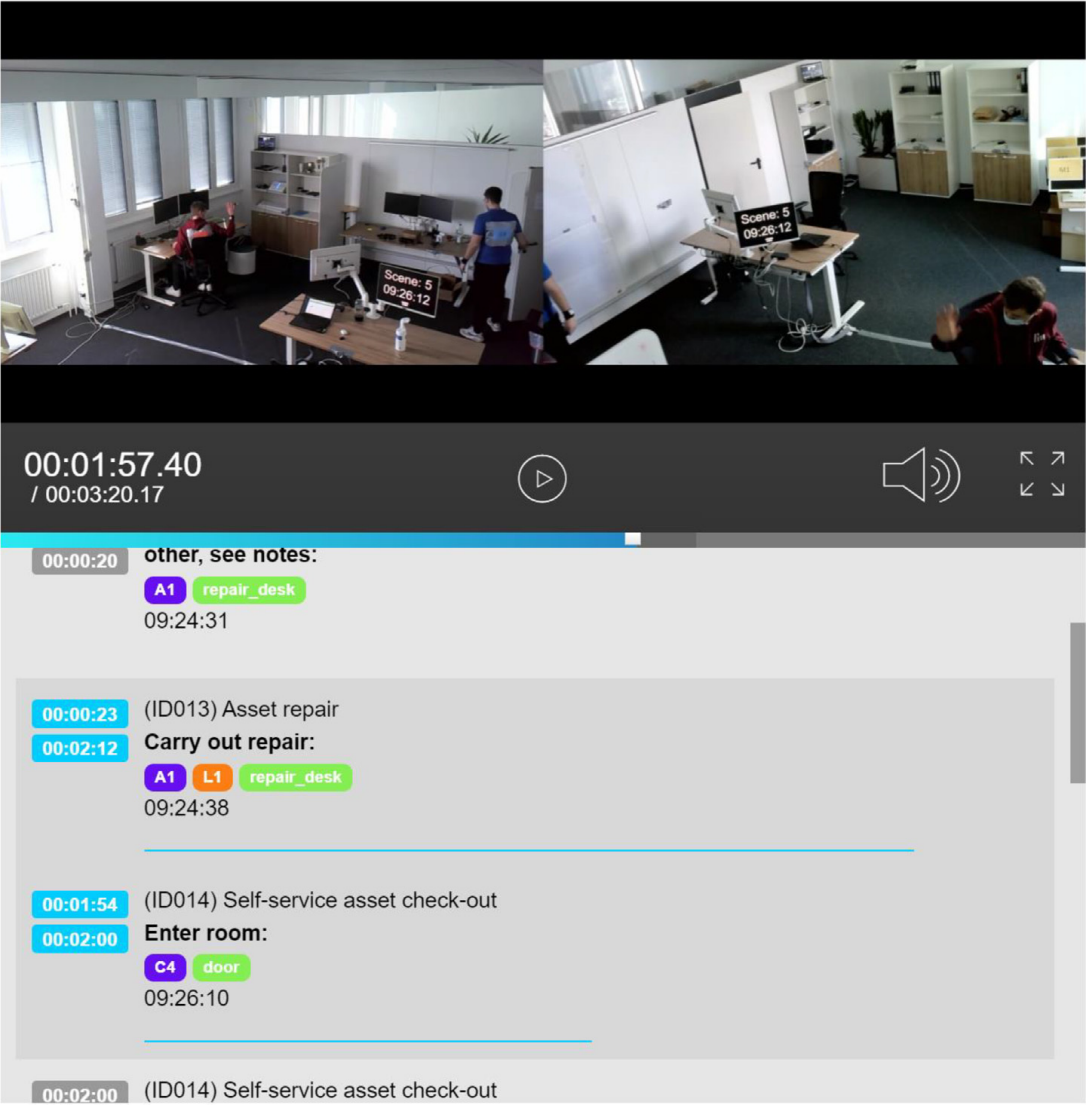
Visualization of a scene with information from *_vid.json and an interactive video player on the dataset website.

The dataset's organization follows a folder-per-scene structure, where each folder (e.g., `scene01/`) mirrors the layout depicted in Table 4. In addition to the scene-level files, the dataset folder `global/` houses the event log file named `itam_ocel.json`. This file captures the events tracked within the ITAM system, formatted in OCEL 1.0 [7]. Next to this file, ‘global_ocel.json’ is the merged log of `itam.jsonocel` and the individual *_ocel.json` files. This file provides the most holistic picture of the dataset. Moreover, beyond the static files in the data repository, the project website features a per-scene metadata-enhanced video player [8] with the annotations from `*_vid.json` as illustrated in Fig. 2.

**Table 4:** Structure of files and their respective fields and features per scene.

Type & File Name	Feature Name	Description
Video *.mp4	–	Raw video in mp4 format. Both camera angles are stacked vertically. A hardcoded timestamp is visible from both camera angles.
Video Log *_vid.json	Offset	Video start timestamp.
	Version	Always ‘1’.
	localisations[]	List of process instances in the scenes containing all subsequent features.
	data.process	Process name.
	data.label	Process instance ID.
	data.variant	Process variant identifier.
	localisation[]	List of segments within a process instance containing all subsequent features.
	tcin	Segment start timestamp.
	tcout	Segment end timestamp.
	level	Always ‘1ʼ.
	activity	Activity name.
	note	(opt) additional notes.
	objects	List of IT asset identifiers (see Table 2*)*
	actors	List of human actor identifiers (see Table 2*)*
	attributes	List of additional attributes related to the activity.
	location	List of location identifiers (see Table 2*)*
*_ocel.json		Joint video and sensor OCEL.
*_video_ocel.json		Video OCEL
*_sensors_ocel.json		Sensor OCEL
Motion Tracking *_mov.csv	Timestamp	Measurement timestamp.
	Resource	Human actor identifier (see Table 2*).*
	Activity	Movement activity (i.e. Walk, Stand, Handling).
	Region	Current area (see Table 2*)*
	walking_speed	Walking speed in m/s.
	step	Indicates if the activity involved a stepping motion.
	handling_height	A numeric value indicating the height at which the resource was handling objects during the activity.
Web Task Mining *_ui.csv	Timestamp	Activity timestamp.
	ExtensionName	Always ‘chrome’.
	eventType	Type of interaction recorded.
	url	Active tab URL.
	tabTitle	Active tab title.
	clipboardText	Clipboard text copied in this activity.
	screenX	Mouse pointer position X value (from top left).
	screenY	Mouse pointer position Y value (from top left).
	keyboardCommand	Keypress value.
	selectedText	Text selected during the event.
	domPath	Hierarchical path of the origin the DOM node.
	version	Always ‘1.0ʼ.
	context.elementType	The HTML element type.
	context.elementId	'id' attribute value of the involved element.
	context.url	The specific URL of the sub-component or frame of the webpage.
	context.elementName	The 'name' attribute of the HTML element.
	context.inputType	For input elements, the type of input.
	context.elementValue	The value property of the element at the time of the event.
	context.className	The 'class' attribute of the element.
	context.checked	Checked value for checkboxes.
	context.x	The x-coordinate of the element on the page.
	context.y	The y-coordinate of the element on the page.
	context.width	The width of the element in pixels.
	context.height	The height of the element in pixels.
	context.extractions	An array of any extra data extracted from the element during the event.
Reed Switches *_reed_switches.csv	time	State change timestamp.
	reed_switch	Sensor location identifier.
	reed_switch_state	Reed switch state change. Either opened or closed.
Distance Sensor Laptop Shelf *_distance_sensor_laptop_shelf.csv	time	Measurement timestamp.
	distance_in_cm	Distance (in cm) from the upper shelf floor to the surface (uppermost laptops on the shelf). This can be used to determine the number of laptops present or detect if a laptop has been taken from or returned to the shelf.
Room Climate *_temp_hum_sensor_window.csv	time	Measurement timestamp
	temp_in_celsius	Room temperature in °C.
	hum_in_percent	Relative room humidity in percent.

## 5 Experimental Design, Materials and Methods

Data were recorded following a script on a scene level, prescribing the involved process variants, IT assets, actors, and planned process deviations (e.g., theft, ignored activities). In planning the data recording schedule, the frequency of non-deviant process instances was prioritized over process instances that demonstrate deviances. The laboratory environment consisted of a single room equipped with sensors. IT assets and human actors are labelled for visual recognition in the scenes.

### 5.1 IT asset selection and labeling

The processes selected for inclusion in this dataset represent typical IT asset management activities encountered in corporate settings. These processes encompass the entire lifecycle of IT assets, from acquisition and deployment to maintenance and eventual decommissioning. The selection of IT assets such as laptops, monitors, mice, headsets, keyboards, and web cameras was strategic, designed to reflect a range of typical interactions that IT staff and clients encounter in a realistic IT asset management setting.

Data labelling in this dataset was executed with high precision to facilitate detailed process analysis. Each asset and human actor involved in the dataset was assigned an identifier, ensuring traceability and consistency across all data types, from video recordings to sensor data and system logs. Here is how the labelling was structured:

• IT Assets: Every asset, including laptops, monitors, and peripherals, was labelled with a unique identifier. This meticulous labelling allows for precise tracking of each asset as it moves through various processes and interactions within the IT environment.
• Human Actors: Each participant in the dataset, representing IT staff and clients, was assigned a code (e.g., A1, C1) to anonymize and track their interactions with the IT assets and systems.
• Environmental Setup: The laboratory environment was divided into specific areas (e.g., warehouse area, working area) and specific locations within these areas (e.g., repair desk, laptop shelf, door), each labelled to associate the actors' and assets' movements and activities with particular locations.

### 5.2 Data collection and preprocessing

**Fig. 3:**
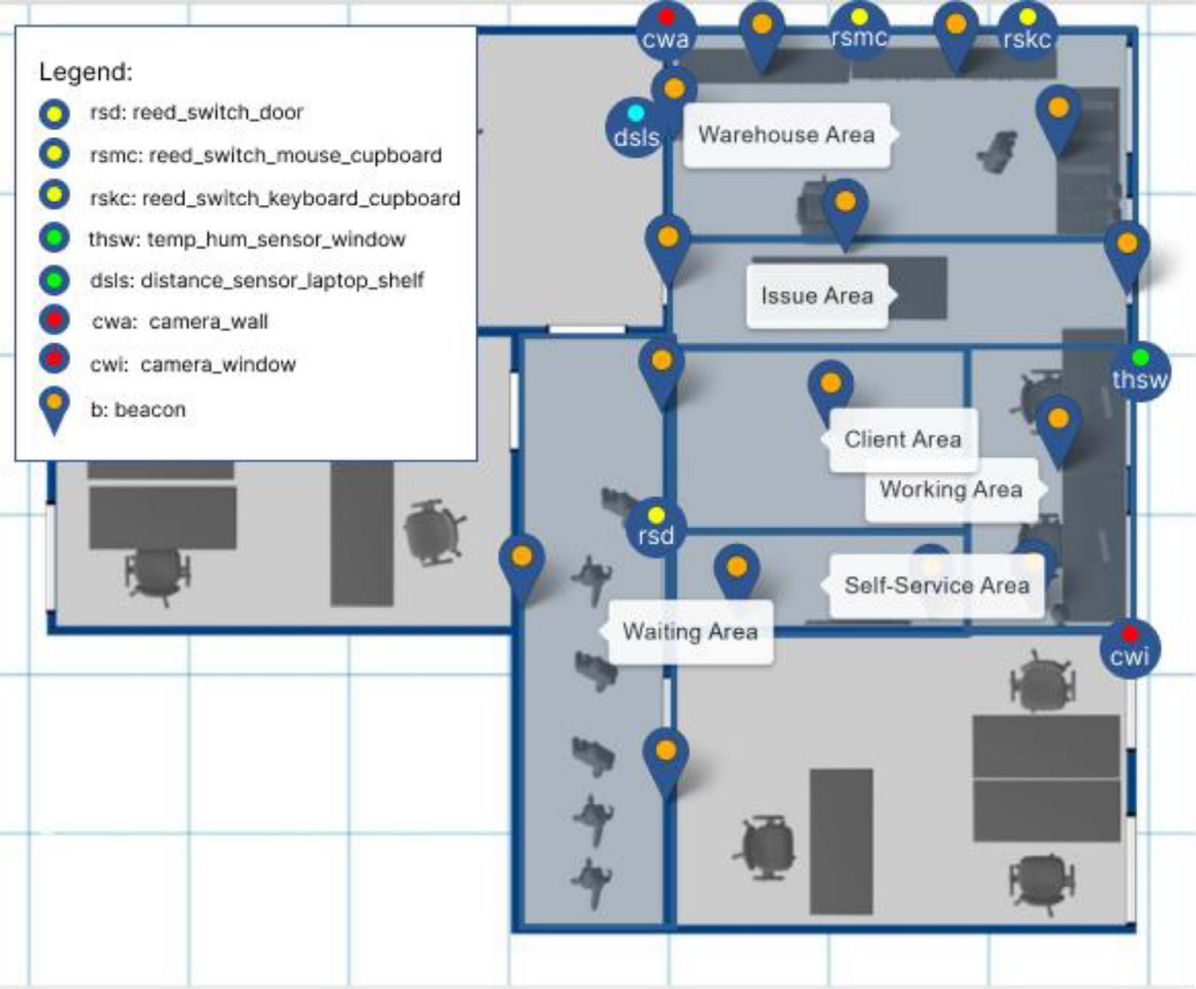
Room plan with areas and sensor positions.

The data collection and preprocessing phase was designed in a certain way to capture a detailed and comprehensive dataset using cameras and a variety of sensors strategically placed to monitor key environmental- and interaction metrics within the designated locations. The room measures 5.50 m by 7.50 m. Fig. 3 gives an overview of the placement of each camera and sensor. These are positioned to optimize data collection for process mining analysis. The zero position of the room, used as a reference for all measurements, is defined as the left bottom corner behind the entrance door.

#### 5.2.1 Ambient sensors and video cameras

To facilitate process mining analysis on video and sensor data, several cameras and ambient sensors were installed in the room to provide video data and both discrete and continuous sensor data. Table 5 gives an overview of the camera and sensor positions. The two cameras were positioned in opposite corners. Each sensor was connected to a separate Raspberry Pi 3B+. The detailled Python scripts used for collecting the sensor data can be found on our Solve4X Project Website. We used the following ambient sensors:

• Reed switches were installed on the room door and on cupboard doors to monitor when they were opened and closed. This setup helps track access to stored IT assets. An event was recorded every time the status of the switch changed from close to open or vice versa.To ensure consistent measuring, the sensor setup was thoroughly tested before each recording, i.e., doors were opened and closed manually and the recorded data was analyzed before starting the live-data collection.
• An ultrasonic distance sensor (Model: HC-SR04) was mounted inside a shelf above a laptop stack. This sensor measured the stack height, providing real-time data on the inventory level. The Python script on the microcontroller was designed to record a distance once per second. However, the time between the two consecutive measurements varies in reality. Here again the setup was tested thoroughly before each recording by manually adjusting the stack height to create a predefined distance to the sensor and by analyzing the resulting measurements. For technical reasons and due to the production process, the sensor measures accurately to fractions of a centimeter, which is sufficient for our use case.
• Temperature and humidity sensor (Model: DHT11): Located at a higher position near the window to measure environmental conditions, which could affect both device performance and optimal storage conditions. The Python script for this sensor was designed to record a temperature and humidity pair every five seconds. However, the time between two consecutive measurements also varies in reality. The sensor was again tested thoroughly before each recording by analyzing measured temperature and humidity values before the live-data collection.

**Table 5:** Sensor overview and placement.

Sensor	Description	Position (x, y, z) (m)
reed_switch_door	Model: Reed switch Type: Discrete sensor Location: Door	0.0, 1.18, 2.03
reed_switch_mouse_cupboard	Model: Reed switch Type: Discrete sensor Location: Mouse cupboard	1.18, 7.50, 1.17
reed_switch_keyboard_cupboard	Model: Reed switch Type: Discrete sensor Location: Keyboard cupboard	2.03, 7.50, 1.17
distance_sensor_laptop_shelf	Model: Ultrasonic distance sensor HC-SR04 Type: Continuous sensor Location: Laptop shelf	0.0, 6.38, 1.52
temp_hum_sensor_window	Model: Temperature and humidity sensor DHT11 Type: Continuous sensor Location: Window	5.50, 3.75, 1.84
camera_window USB camera attached to a laptop	Model: Verbatim AWC-02 Full HD Location: Window corner Recording 1280 × 720 (30fps)	5.50, 0.0, 2.92
camera_wall USB camera attached to a laptop	Model: Verbatim AWC-02 Full HD Location: Wall corner Recording: 1280 × 720 (30fps)	0.0, 7.50, 2.92
Beacons	For motion tracking	See Fig. 3

#### 5.2.2 IT asset management system

**Fig. 4:**
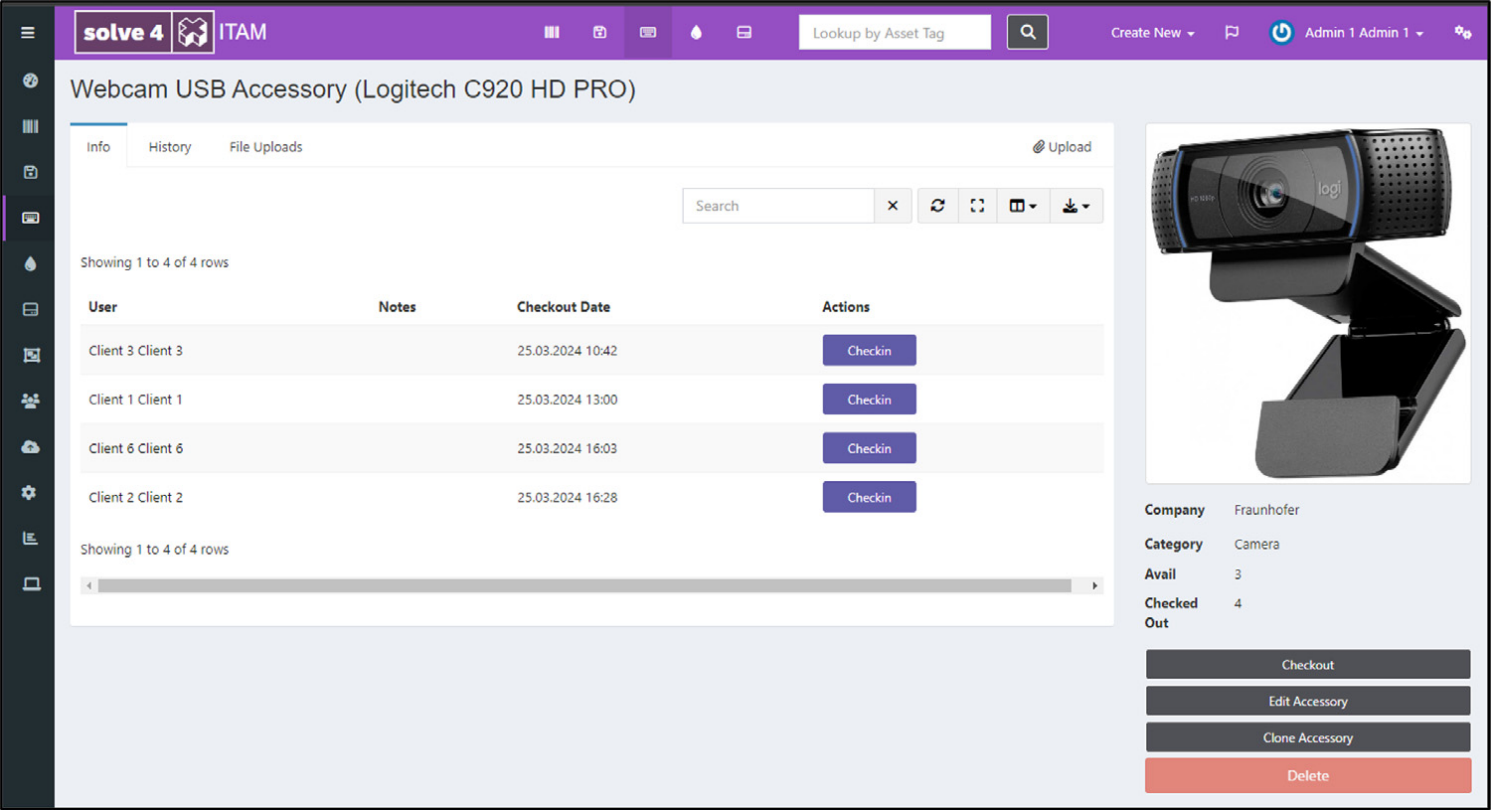
Screenshot of the ITAM system showing an accessory page from which users can check out available webcams.

*ITAM activity recording.* The open-source ITAM system Snipe-IT^2^ was installed and configured for the experiment. Assets and client accounts were created upfront. The system features all the functionality needed to manage IT assets for use in the planned ITAM processes in a web interface accessible to IT staff and clients. Both user roles can log into the system and perform process activities in sync with those visible on the video. All transactional activities involving IT asset status changes are recorded in the ITAM system's database during the data acquisition experiment. Fig. 4 depicts a screenshot of the ITAM system.

**Fig. 5:**
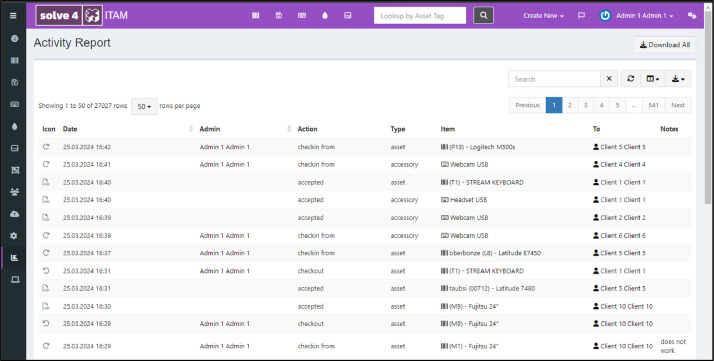
Screenshot of the ITAM system showing the overall activity report.

*Data Processing.* After the data recording, the transactional events were exported and transformed into an OCEL 1.0 event log file [7] using a custom library.^3^ The ITAM system keeps track of all relevant activities and involved objects by design (see Fig. 5).

#### 5.2.3 Motion tracking

*Motion recording.* Human actor movement data was recorded using technology offered by MotionMiners,^4^ a technology provider for analyzing manual work processes. The planning, placement of measurement beacons, and the definition of the areas in the room influence the measurement accuracy. Areas must be planned without overlap. The beacons were mounted to fixed points at 80–90 cm height. For calibration, we followed instructions provided by the tool vendor: Using a mobile phone and equipped with a set of measurement devices, a author walks each area, performing various gestures and positions that may be relevant for later recoding. This procedure is repeated three times. Raw data from motion tracking was recorded for scenes 1–15 only. Each human actor was equipped with three sensors, attached to the wrists and the belt, continuously recording movement data and position information after the room was equipped and calibrated with wireless beacons.

*Data Processing.* After the data recording, motion tracking data was exported from the individual wearable sensors and consolidated per set (i.e., two wrist sensors and one belt sensor). The raw data is stored granularly (20 events/second). In a processing step, the events were aggregated to one-second intervals. In an additional processing step, the technical Set-IDs were mapped to the human actor IDs as references throughout the dataset. This mapping was semi-automatically performed using the scene script containing the information about which human actor is wearing a particular motion tracking sensor set in each scene.

#### 5.2.4 Web task mining

*Web task recording.* User interaction logs help analyze business processes on a task basis (the individual building parts of a process activity) [3]. The IT staff laptop was equipped with a custom browser plugin to capture interactions within the web browser. The plugin records detailed actions such as mouse clicks and keystrokes.

*Data Processing.* After the data recording, the user interaction log was split into individual files per scene.

#### 5.2.5 Video data collection and processing

*Video Recording.* The project involved recording video data from two opposite camera angles to cover all activities fully. The recordings were synchronized and segmented into individual scenes. To achieve a comparable appearance of the videos and avoid disturbances, each camera's autofocus was disabled and artificial lightning was used to maintain brightness. Videos were processed by cutting raw material into 2 × 36 individual, pairwise frame-exact, scene files. The processing was done using LosslessCut (v3.59.1.0),^5^ a graphical user interface for the FFmpeg^6^ library for lossless operations on media files. Also, pairs of videos for the same scene were vertically stacked using FFmpeg. These stacked video files are deposited in the dataset.

**Fig. 6:**
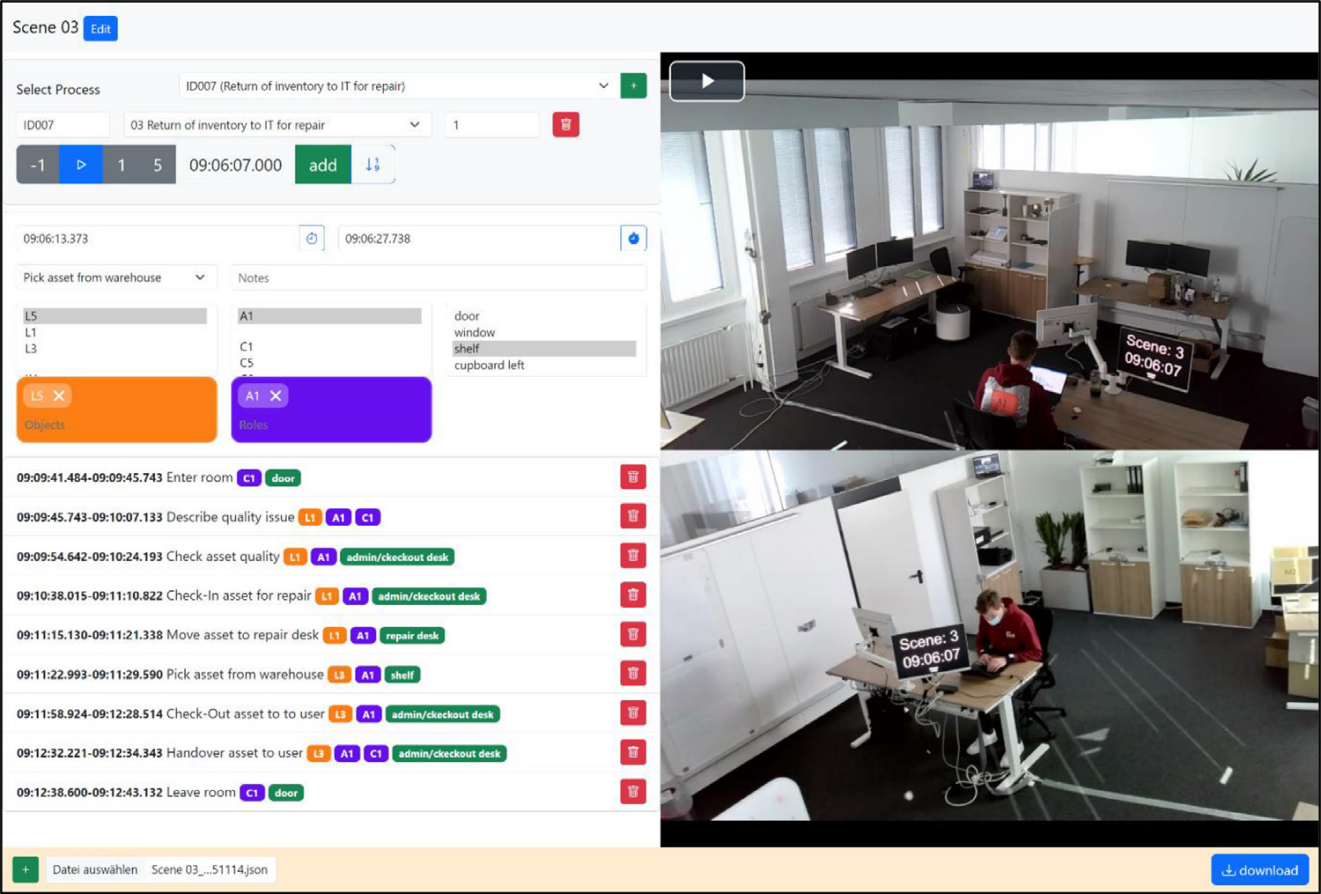
Video annotation with process instances and segments representing process activities.

*Video Annotation.* After processing the raw video, the author team manually annotated business process activities using a custom tool designed and built for the project [9] (see Fig. 6). The web application is initialized with a configuration file per scene to identify the video file location and predetermined scenes as well as involved actors to prefil selection menus for faster editing. Users can then choose a scene for annotation and play the video on the right side of the application. On the left side, all segments within a scene are listed for a quick overview. New segments can be added by clicking a button, and the current position in the video is used as the start point of the activity. The end timestamp can be selected by seeking the end of the segment in the video player. Users select the activity, objects involved, actors, and location from the menus and save the segment. After aligning on a coding style within the author team (i.e., what activities should be coded, when objects, human actors, and locations are coded), the tool was helpful in quickly and accurately annotating the videos in parallel. The annotation files could be downloaded for further processing and imported again for corrections. With the annotations, we provide ground truth for activity detection and process mining analysis (Table 1).

## Limitations

Motion tracking data is only available for Scene 01 – Scene 15. A short setup was required for most scenes to ensure equipment was in place and human actors could change roles. Hence, all data except for data from the ITAM system were cut to match the exact intervals recorded on video. Therefore, concatenation of data does not lead to a continuous picture. While any efforts were made to ensure continuity regarding the allocation and state of IT assets, ambient sensors might expose notable jumps in data from scene to scene (e.g., a physical laptop was placed on the laptop shelf in a setup period to being able to issue said laptop in the following scene, leading to an unexplained jump in the distance sensor data). Further, the ITAM lifecycle and processes were simplified to generate many process instances (e.g., individuals should not pick up four laptops and monitors daily and return them for repair).

## Ethics Statement

We obtained informed consent from all participants for their participation in this dataset collection and publication of anonymous data.

## CRediT authorship contribution statement

**Diana Chvirova:** Conceptualization, Investigation, Writing – original draft. **Andreas Egger:** Conceptualization, Methodology, Software, Investigation, Data curation, Writing – review & editing. **Tobias Fehrer:** Conceptualization, Methodology, Software, Investigation, Data curation, Writing – original draft, Writing – review & editing. **Wolfgang Kratsch:** Writing – review & editing. **Maximilian Röglinger:** Writing – review & editing. **Jakob Wittmann:** Conceptualization, Investigation, Data curation, Writing – review & editing. **Niklas Wördehoff:** Conceptualization, Investigation, Data curation, Writing – review & editing.

## Data Availability

Business Processes in IT Asset Management Multimedia Event Log (Original data). (Figshare)

## Footnotes

1 Solve4X Project Website: https://x2log.pages.fraunhofer.de/solve4x.

2 Snipe-IT: https://snipeitapp.com/.

3 Tobias Fehrer, Laravel EventLog, Github, last changed 08.03.2024, https://github.com/dtdi/laravel-eventlog.

4 MotionMiners, last accessed 22.04.2024, https://www.motionminers.com/.

5 LosslessCut: https://github.com/mifi/lossless-cut.

6 FFmpeg: https://ffmpeg.org/.

## References

[1] van der Aalst W., Reinkemeyer L. (2020). Process Mining in Action.

[2] Kratsch W., König F., Röglinger M. (2022). Shedding light on blind spots – developing a reference architecture to leverage video data for process mining. Decis. Support Syst..

[3] Abb L., Rehse J.-R. (2024). Process-related user interaction logs: state of the art, reference model, and object-centric implementation. Inf. Syst..

[4] Kecht C., Egger A., Kratsch W., Röglinger M. (2023). Quantifying chatbots’ ability to learn business processes. Inf. Syst..

[5] Egger A., ter Hofstede A.H.M., Kratsch W., Leemans S.J.J., Röglinger M., Wynn M.T., Dobbie G., Frank U., Kappel G., Liddle S.W., Mayr H.C. (2020). Conceptual Modeling.

[6] T. Fehrer, A. Egger, D. Chvirova, J. Wittmann, N. Wördehoff, W. Kratsch, M. Röglinger, Business processes in IT asset management multimedia event log, figshare, 2024. 10.1016/j.dib.2024.110716 PMC11305206 39113786

[7] Ghahfarokhi A.F., Park G., Berti A., van der Aalst W.M.P., Bellatreche L., Dumas M., Karras P., Matulevičius R., Awad A., Weidlich M. (2021). New Trends in Database and Information Systems.

[8] Hervé N., Letessier P., Derval M., Nabi H. (2015). Proceedings of the 23rd Annual ACM Conference on Multimedia Conference.

[9] T. Fehrer, process-aware-video-annotator. https://github.com/dtdi/process-aware-video-annotator, 2024.

